# Twin‐peg and single‐peg unicompartmental knee arthroplasty designs show comparable clinical outcomes and radiographic results: A systematic review

**DOI:** 10.1002/jeo2.70290

**Published:** 2025-06-05

**Authors:** Sadra Mohebbi, Yashar Khani, Elias Sadooghi Rad, Mohammadhossein Hefzosseheh, Mahya Aliakbari, Fateme Rezagholi, Amir Mehrvar

**Affiliations:** ^1^ School of Medicine Tehran University of Medical Sciences Tehran Iran; ^2^ Student Research Committee, School of Medicine Shahid Beheshti University of Medical Sciences Tehran Iran; ^3^ Student Research Committee, Faculty of Medicine Birjand University of Medical Sciences Birjand Iran; ^4^ Student Research Committee Shiraz University of Medical Sciences Shiraz Iran; ^5^ Student Research Committee Bushehr University of Medical Sciences Bushehr Iran; ^6^ Faculty of Medicine Qazvin University of Medical Sciences Qazvin Iran; ^7^ Clinical Research Development Units, Taleghani Hospital Shahid Beheshti University of Medical Sciences Tehran Iran

**Keywords:** single‐peg, twin‐peg, UKA, unicompartmental knee arthroplasty

## Abstract

**Purpose:**

This systematic review compares single‐peg and twin‐peg unicompartmental knee arthroplasty (UKA) implant designs with respect to radiographic findings, clinical outcomes and implant longevity.

**Methods:**

A search strategy was applied to four databases, including PubMed, Scopus, Embase, and Web of Science. Inclusion criteria focused on studies of the twin‐peg and single‐peg designs in the UKA. Two reviewers independently performed screening, data extraction, and quality assessment. Study characteristics, patient demographics, clinical outcomes, revision rate and radiographic differences were extracted. We utilized the Risk of Bias (RoB) 2 tool and Risk of Bias in Non‐Randomized Studies of Intervention to assess the RoB in included studies.

**Results:**

Seven studies were included in the final review. Results varied in the case of radiographic scores, clinical outcomes and patient‐reported outcome measures (PROMs). Some studies demonstrated advantages for twin‐peg designs in component positioning, but others identified no significant differences. PROMs indicated improvements with both implants, but there was no evidence to clearly prefer one design over the other. Twin‐peg designs showed slightly lower revision rates in some studies, but evidence remains inconclusive regarding overall superiority. Revision causes included pain, osteoarthritis (OA), instability, infection and aseptic loosening.

**Conclusions:**

The study found that twin‐peg designs may have superior outcomes, but overall results did not support either design, conclusively.

**Level of Evidence:**

Level III, systematic review.

**Abbreviations**
AKS‐F: american knee society functional
DXA: dual‐energy X‐ray absorptiometry
HSS: hospital for special surgery
KOOS: knee osteoarthritis outcome score
KSS: knee society score
OA: osteoarthritis
OKS: oxford knee score
PRISMA: preferred reporting items for systematic reviews and meta‐analysis
PROM: patient‐reported outcome measure
RCT: randomized controlled trial
RoB: risk of bias
ROBINS: risk of bias in non‐randomized studies of intervention
Robvis: risk‐of‐bias visualization
ROM: range of knee motion
RSA: radiostereometric analysis
SF‐36: 36‐Item Short‐Form Survey
TKA: total knee arthroplasty
UKA: unicompartmental knee arthroplasty
WOMAC: western ontario and mcmaster universities osteoarthritis score

## INTRODUCTION

Total knee arthroplasty (TKA) is a reliable surgical procedure that can relieve pain and improve the function of patients with end‐stage knee osteoarthritis (OA) [1, 8, 29]. Although TKA has demonstrated great outcomes, such as implant survival rates greater than 90%, the procedure is associated with some complications, including aseptic loosening, fracture, instability, and periprosthetic joint infection [3, 20]. Notable improvements in the implant design and technique of unicompartmental knee arthroplasty (UKA) have led to increasing attention in research and clinical practice. Therefore, UKA, only affecting one compartment of the knee, has recently been introduced as a successful and viable treatment for arthritis [18]. On the other hand, some surgeons remain sceptical about the durability and efficacy of this treatment, considering TKA the gold standard treatment for arthritis.

UKA has shown better kinematics and good long‐term survivorship in many studies; however, the main problem with this technique is its higher revision rates compared to TKA [6, 13, 18]. The higher revision rates have mostly been reported due to aseptic loosening in a Norwegian study [4]. According to data from various studies, aseptic loosening seems to be a global problem, mostly affecting the femoral component of the implant [4, 7]. One of the most frequently used UKA implants is the Oxford UKA (Zimmer Biomet) [23]. The femoral component in the Oxford Phase III implant has a spherical shape, with one anchoring peg inserting into the femoral condyle [23]. In 2003, in an attempt to improve the stable fixation and address the loosening problem, the new Oxford Partial implant was introduced with a twin‐peg design. In order to support the extra peg in this new design, they advanced the femoral component 15° to the anterior, providing further support as a result of the larger surface area [2].

The twin‐peg femoral component is associated with a high‐quality outcome and a low incidence of complications, as evidenced by White et al. However, it has not yet been established how implant design changes, surgical expertise, and surgical technique significantly affect the risk of developing long‐term complications when using the twin‐peg Oxford implant [26, 27, 28]. There are a few mid‐ to long‐term follow‐up studies comparing the single‐peg design and the twin‐peg implants. No previous systematic review has compared clinical outcomes of single‐peg and twin‐peg implant designs, and therefore it is uncertain whether there could be any advantage in terms of outcome to the patient. This study aims to provide a comprehensive, systematic review of the literature. It compares clinical and patient‐reported outcome measures (PROMs) between the twin‐peg and single‐peg UKA implant designs. We hypothesized that the twin‐peg UKA implant designs have better clinical outcomes compared to single‐peg designs.

## MATERIALS AND METHODS

### Search strategy

This study was conducted based on Preferred Reporting Items for Systematic Reviews and Meta‐analyses (PRISMA) guidelines [19]. The study protocol was pre‐registered on the Open Science Framework using an a priori approach at (https://osf.io/bufyx/). A search strategy was applied to four databases, PubMed, Scopus, Embase and Web of Science, using MeSH keywords. All relevant papers were searched without language restrictions and translated if necessary. The search strategy was as follows: (‘unicompartmental knee arthroplasty’ OR ‘unicompartmental knee replacement’ OR ‘Unicondylar knee arthroplasty’ OR ‘Unicondylar knee replacement’ OR ‘UKA’ OR ‘UKR’) AND (‘twin‐peg’ OR ‘twin‐pegged’ OR ‘twin peg’ OR ‘twin pegged’ OR ‘2‐peg’ OR ‘peg’).

### Inclusion and exclusion criteria

Studies were only included if they had compared the twin‐peg with single‐peg designs in patients who had undergone UKA. Studies that performed UKA in human cadaver knees were excluded.

### Study selection

Two investigators (SM and YK) independently screened the search results, and studies that were not relevant were excluded. Discrepancies in the included studies were resolved through discussions among the reviewers.

### Data extraction

For data extraction, data from all included studies were independently extracted by two investigators (SM and YK) using a pre‐established data extraction form, and any disagreements were resolved through discussion. Study characteristics, such as first author, year of publication, country and study type, were included as part of the extracted data. Furthermore, participant‐related information such as age distribution, gender composition, and the number of knees in each group were recorded. Follow‐up duration and outcome measures were also among the extracted information.

### Risk of Bias (RoB) assessment

We evaluated randomized controlled trials (RCTs) using the Cochrane RoB 2 tool [25]. The Risk of Bias in Non‐Randomized Studies of Intervention (ROBINS‐I) tool was used to evaluate non‐RCTs (non‐RCTs) [24]. The bias assessment report was visualized using the Risk‐of‐Bias Visualization (Robvis) tool [14].

## RESULTS

### Selection process of studies

**Figure 1:**
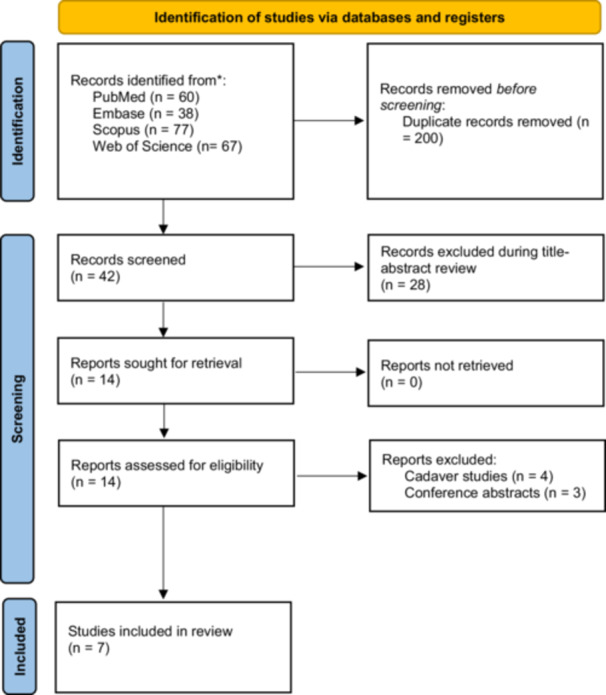
PRISMA flow diagram. PRISMA, Preferred Reporting Items for Systematic Reviews and Meta‐Analysis.

After conducting a search strategy in databases, a total of 242 articles were initially found. After the removal of duplicate articles, 42 unique studies remained. Only studies that compared the single‐peg and twin‐peg implants were retained during title and abstract screening. Following this screening, 14 articles that met the inclusion criteria were proceeded to full‐text review. Finally, full‐text screening was conducted to select studies that met all inclusion criteria. Three studies were conference abstracts, four were cadaveric studies, which were excluded, and ultimately, seven studies were included in this systematic review (Figure 1).

### Study characteristics

The study designs in the seven final articles varied, with two RCTs and five cohort studies [9, 10, 15, 16, 23, 27, 30]. Outcome measures assessed in the studies included implant survival and revision, radiographic differences, as well as patient‐reported outcome questionnaires such as the Oxford Knee Score (OKS), American Knee Society Functional (AKS‐F) score, Western Ontario and McMaster Universities Osteoarthritis score (WOMAC), the Knee Society Score (KSS) and Hospital for Special Surgery (HSS). The study characteristics of these seven final articles included in the systematic review study are summarized in Table 1.

**Table 1:** Characteristics of the included studies.

					Participants				
Author/year	Country	Study type	Mean age (years)	Sex (female)	Total	Single‐peg	Twin‐peg	Follow‐up (years)	Outcome measures
Horsager et al. [9] (2019)	Denmark, Netherlands	RCT	63.62	32 (40%)	80	29	51	5	Wear‐rate comparison OKS
Hurst et al. [10] (2014)	USA	Cohort	63.5	192 (53%)	413	223	190	N/A	Radiographic differences
Mohammad et al. [15] (2020)	United Kingdom	Cohort	65	2869 (50%)	5668	2834	2834	3.3	Implant Survival Implant revision
Mosegaard et al. [16] (2023)	Denmark	RCT	63.45	32 (40%)	80	29	51	5	Radiographic differences RSA DXA SF‐36 KOOS Implant revision
Skaden et al. [23] (2023)	Norway	Cohort	65.8	3551 (47.7%)	7444	908	6536	8	Implant Survival Implant revision
White et al. [27] (2015)	United Kingdom	Cohort	67	121 (48.5%)	249 patients (288 implants)	0	288	5.1	Implant Survival, OKS score AKS‐F score Patient Satisfaction, The Tegner Activity score Knee ROM
Zhao et al. [30] (2018)	China	Cohort	63.11	19 (73%)	26	15	11	2.1	Radiographic differences HSS score WOMAC score OKS score KSS score

Abbreviations: AKS‐F, american knee society functional score; DXA, dual‑energy X‑ray absorptiometry; HSS, hospital for special surgery; KOOS, knee osteoarthritis outcome score; KSS, knee society score; OKS, oxford knee score; RCT, randomized controlled trial; ROM, range of knee motion; RSA, radiostereometric analysis; SF‐36, 36‐item short form survey; WOMAC, western ontario and mcmaster universities osteoarthritis index.

### RoB of included studies

**Figure 2:**
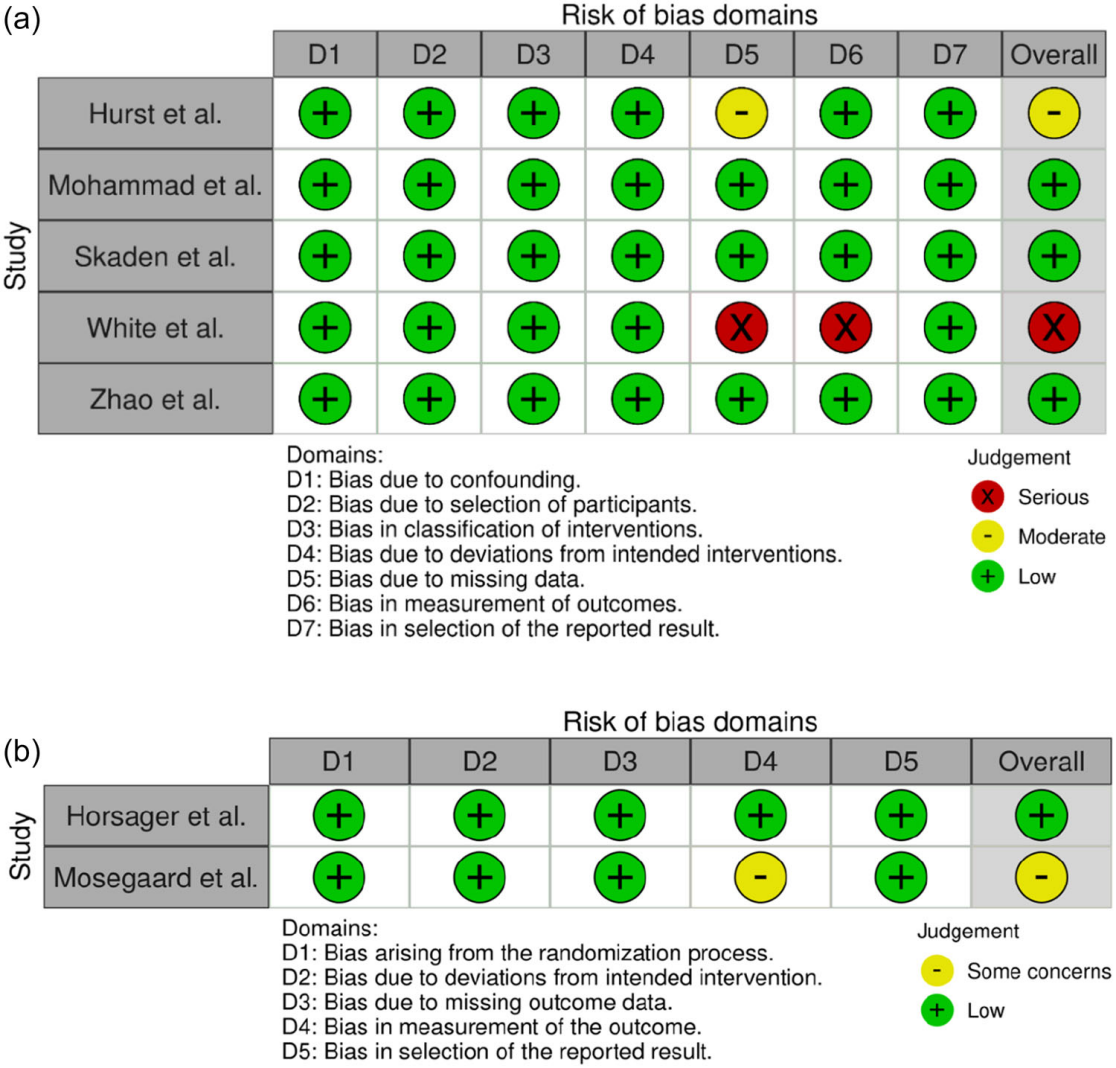
Risk of bias (RoB) assessment based on ROBINS‐I (a) and RoB‐2 (b). ROBINS‐I, risk of bias in non‐randomized studies of intervention.

The RoB evaluation of the studies using the ROBINS‐I and ROB‐2 checklists demonstrated four low RoB, two moderate RoB and one severe RoB. The reason for this severe RoB in this study was the external control group and the reason for the moderate RoB in two studies was inadequate strategies to manage the missing data. The bias assessment report was illustrated in Figure 2. This shows an overall medium‐quality study.

### Radiographic comparison

Three studies evaluated radiographic scores and measurements in single‐peg and twin‐peg UKA [10, 16, 30]. In these three studies, positioning in the femoral component was the most commonly assessed. Besides that, Hurst et al. assessed alignment in the tibial component [10]. Correlations between functional scores and prosthesis position were also evaluated in the Mosegaard et al. and Zhao et al. studies [16, 30]. Although Hurst et al. [10] demonstrated superior alignment in twin‐peg design, Mosegaard et al. [16] and Zhao et al. [30] found no significant difference in radiological scores.

### Clinical outcomes

Seven studies were included in this study. Studies varied in the assessed scores, and some used several PROMs. OKS was the most commonly used PROM in the four studies [9, 16, 27, 30]. Besides OKS, White et al. reported AKS‐F, range of knee motion (ROM), and the Tegner Activity [27], Zhao et al. reported HSS, WOMAC, and KSS [30], and Mosegaard et al. reported KOOS and SF‐36 [16]. Overall results in regard to PROMs were summarized in Table 2.

**Table 2:** Clinical outcome comparison of single‐peg and twin‐peg implants.

Study	Clinical outcomes
Horsager et al. [9] (2019)	The twin‐peg implant demonstrated a better improvement in OKS than the single‐peg, but it was not statistically significant.
White et al. [27] (2015)	The twin‐peg knee had better clinical outcomes than the single‐peg knee and offered a high degree of sustained patient satisfaction
Zhao et al. [30] (2018)	The single‐peg implant demonstrated a better HSS, WOMAC and KSS, but there was no statistically significant difference in the radiological scores and functional scores between the double‐peg and single‐peg implants.
Mosegaard et al. [16] (2023)	SF‐36 and KOOS improvements were similar between single‐peg and twin‐peg designs, and twin‐peg was not superior to the single‐peg design.

Abbreviations: HSS, hospital for special purposes; KOOS, knee osteoarthritis outcome score; KSS, knee society score; OKS, oxford knee score; ROM, range of knee motion; SF‐36, 36‐item short form survey; WOMAC, western ontario and mcmaster universities osteoarthritis.

### Implant performance

The comparison of single‐peg and twin‐peg implants shows varying survival and revision rates. The survival of implants was illustrated in three studies [15, 23, 27]. In these studies, excellent survival of twin‐peg prostheses was reported. However, Skaden found no significant differences between the two designs 5 years after the operation [16]. The revision rate was reported in four studies, and data for 638 revisions were provided [15, 16, 23, 27]. Major causes of revision included pain, OA progression, instability, infection, luxation, malalignment and aseptic loosening, which included at least 70% of all revision causes in all cases in this study. Revision rates and major revision causes are presented in Tables 3 and 4. Twin‐peg implants have marginally lower revision rates.

**Table 3:** Revision rates and survivorship of knee implants.

	Revision			Survival (%)			
Author, year	Total	Twin‐peg, *n* (%)	Single‐peg, *n* (%)	Twin‐peg	Single‐peg	Follow‐up (years)	Note
	602						
White et al. [27], 2015	5	5 (1.7)	NA	98	NA	9	–
Mohammad et al. [15], 2019	230	99 (3.5)	131 (5.4)	96.2	94.8	5	Sig
Mosegard et al. [16], 2022	2	1 (2.0)	1 (3.4)	NA	NA	–	–
Skaden et al. [23], 2022	365	294 (4.5)	71 (7.8)	94	92	5	Not sig

**Table 4:** Revision reasons.

	White (2015)		Mohammad (2019)		Mosegard (2022)		Skaden (2022)	
	Twin (%)	Single (%)	Twin (%)	Single (%)	Twin (%)	Single (%)	Twin (%)	Single (%)
Pain	33.3	–	13.4	23.2	–	–	23.5	31.3
Osteoarthritis progression	50	–	37.3	26.2	–	–	15.5	16.7
Instability	16.7	–	10.4	6.1	–	–	15	12.5
Infection	–	–	7.5	7.1	–	–	15.2	5.2
Aseptic loosening	–	–	22.4	21.2	100	–	13.4	18.8
Luxation	–	–	6	8.1	–	100	10.7	6.2
malalignment	–	–	3	8.1	–	–	6.7	9.3
Total (*n*)	6	–	67	99	1	1	328	96

## DISCUSSION

The most important findings of this systematic review are that twin‐peg and single‐peg UKA designs demonstrate comparable clinical outcomes, PROMs and radiographic results, with no conclusive evidence supporting the superiority of one design over the other. While some studies suggested advantages for twin‐peg designs regarding component positioning and slightly lower revision rates, these differences were inconsistent across all included studies.

The investigations of radiographic outcomes in Mosegaard et al. and Zhao et al. revealed no significant differences in femoral component positioning between the single‐peg and twin‐peg designs [16, 30], but Horsager et al. showed a significant difference [9]. The reason for this controversy is that Horsager et al. measured radiographic outcomes in the short term, but the other two studies had mid‐ to long‐term follow‐up times. This is consistent with findings of previous research by D'Ambrosi et al., which found that design modifications did not significantly affect radiographic outcomes [2].

Clinical outcomes evaluated using various PROMs showed mixed findings. In some studies, such as those conducted by Horsager et al., Zhao et al. and Mosegaard et al., some improvements in twin‐peg implants compared to single‐peg implants were reported, but they were not statistically significant. On the other hand, White et al. indicated significant improvements in clinical scores, but the possible reason for that is an external single‐peg group as the control group, which may result in some bias [27]. This aligns with the findings by Murray and Parkinson, who highlighted that while newer implant designs show promise, they often do not demonstrate substantial clinical benefits over established designs in broad patient populations [18].

The twin‐peg design offered a slight advantage in terms of implant survivorship and revision rates. Multiple included studies reported lower revision rates for twin‐peg implants, and Mohammad et al. found significant differences in the revision rate between these two implants [15]. In contrast, Skaden et al. did not find statistically significant differences in survival rates. This indicates that while the twin‐peg design could have some advantages, it does not always result in improved clinical performance [23]. Differences in surgical technique, patient demographics, and follow‐up time in the studies can explain this different outcome.

According to the RoB assessment, many included studies categorized as moderate quality, but the methodological heterogeneities, such as differences in study designs, patient populations, outcome measures, scoring systems and follow‐up durations, made it difficult to reach a definitive conclusion. Also, in many primary studies, the differences between these two implant designs in terms of different scores and other clinical outcomes were not identified as significant, which indicates that other variables besides the implant design may influence patient outcomes. Generally, in different studies, the twin‐peg design has seemed to have some advantages and also lower revision rates compared to the single‐peg design, but the improved clinical outcomes are not consistent for all individuals regarding clinical improvements. Therefore, surgeons need to consider patient‐specific variables, such as preoperative knee function, activity level and comorbidities, when choosing between single‐peg and twin‐peg implant designs. Also, future research should be conducted with a longer follow‐up duration and RCTs with a larger sample size to increase the certainty of the evidence of the long‐term benefits of the twin‐peg design and optimize UKA outcomes.

Since the 1970s, the cemented Oxford UKA has been in clinical use, yielding good clinical outcomes [17]. The cementless UKA was introduced in an attempt to improve the fixation of the implant and lower the revision rate, which is mainly due to aseptic loosening. The difference between the cementless and cemented implants is the porous titanium having a hydroxyapatite coating, which is placed on a small additional peg on the femoral component [11]. Comparing different implants regarding their fixation method and design at the same time is challenging. Among the seven studies included in this study, three compared twin‐peg designs concerning cement [9, 16, 23]. Comparing the uncemented twin‐peg to the cemented twin‐peg, we discovered a greater chance of revision for periprosthetic fracture, infection and polyethylene wear for the uncemented design [12, 23]. However, Horsager et al. found no significant differences in polyethylene wear between cemented and cementless twin‐peg UKAs and also no comparison was made between cemented or cementless single‐peg designs [9]. Periprosthetic fracture has been reported as the most common cause of revision in the cementless version of twin‐peg [23]. Nevertheless, the results of this study provide the basis for additional studies, including prospective RCTs comparing cementless and cemented implant fixation in each design group and also with each other.

In our study, we only included clinical studies, but the literature also included cadaver studies. To give examples, Reiner conducted two studies in 2014 and 2018 [21, 22]. In the study conducted in 2014, he found no significant difference between the stability of the two designs of femoral components with respect to micromotion or subsidence under cyclical loading. In the study year 2018, it was determined that the maximum load to failure was higher in the twin‐peg group compared to the single‐peg group, which can be explained by the additional peg, which leads to stronger implant fixation at the cement‐bone interface [21, 22]. In Eckert et al.'s 2022 study, the single‐peg showed higher compression at 70° flexion, whereas the twin‐peg design showed higher compression at 115°, and the probable reason for that may be the increasing surface contact and distribution of forces might increase mechanical stability at higher degrees of flexion (115°) with the twin‐peg design. In contrast, the single‐peg design may not distribute forces as effectively, resulting in higher compression at lower flexion angles (70°). Also, X‐displacement was significantly higher for the single‐peg at 115°, which is probably due to the fact that in the case of single‐peg designs, additional anchoring points in the knee structure may be insufficient to provide sufficient stability when the knee is flexed at higher angles [5].

This systematic review helps surgeons choose between twin‐peg and single‐peg UKA designs based on patient‐specific factors and surgical goals. Twin‐peg designs showed slightly better component positioning and lower revision rates, but overall evidence did not strongly favour one design. Both designs provide significant pain relief and functional improvement, with no clear superiority in patient satisfaction. However, surgeons should be cautious about the slightly higher risk of aseptic loosening with single‐peg designs, especially in patients with poor bone quality or requiring long‐term durability.

This systematic review has several limitations that should be acknowledged. First, the included studies exhibited methodological heterogeneity, including variations in study designs, patient demographics, outcome measures, and follow‐up durations, which made it challenging to draw definitive conclusions. Second, the RoB assessment revealed that some studies had moderate to severe RoB, potentially affecting the reliability of the findings. Third, the small number of included studies (*n* = 7) and the lack of long‐term follow‐up data in many of them limited the ability to assess the durability and long‐term performance of the twin‐peg and single‐peg designs. Additionally, the focus on Zimmer Biomet implants may limit the generalizability of the findings to other UKA designs. Finally, the absence of RCTs with large sample sizes further restricts the strength of the evidence.

## CONCLUSION

The study compared single‐peg and twin‐peg UKA designs, finding no clinical differences. While some studies showed twin‐peg designs were better in positioning and alignment, no significant differences were found. Functional outcomes showed improvements in both designs, but no clear preference was found.

## AUTHOR CONTRIBUTIONS

All authors contributed substantially to this study. Also, all authors read and approved the final manuscript. Amir Mehrvar was the supervisor and formed the research question. Study selection, risk of bias assessment and data extraction: Sadra Mohebbi and Yashar Khani. Preparation of the first draft of the paper: Mohammadhossein Hefzosseheh and Mahya Aliakbari. Critical revision of the first draft: Elias Sadooghi Rad and Sadra Mohebbi. Designing the methodological aspects of the study: Fateme Rezagholi and Yashar Khani.

## CONFLICT OF INTEREST STATEMENT

The authors declare no conflicts of interest.

## ETHICS STATEMENT

The ethics statement is not available.

## Data Availability

Data sharing is not applicable to this article, as no data sets were generated or analyzed during the current study.
